# Integrated metabolomic and transcriptomic analysis reveals the effects and mechanisms of Jingfang Baidu powder on acute lung injury

**DOI:** 10.3389/fphar.2025.1649883

**Published:** 2026-01-09

**Authors:** Zimo Zhu, Baimei Cui, Rong Dai, Guoxian Hu, Sue Xu, Xianfang Zhao, Wenjing Wang, Xiufang Li

**Affiliations:** 1 College of Traditional Chinese medicine, Yunnan University of Chinese Medicine, Yunnan, China; 2 School of Chinese Materia Medica and Yunnan Key Laboratory of Southern Medicine Utilization, Yunnan University of Chinese Medicine, Yunnan, China

**Keywords:** acute lung injury, alveolar epithelialbarrier, inflammation, Jingfang Baidu powder, Poly(I:C)

## Abstract

**Background:**

Acute lung injury (ALI) is one of the most prevalent respiratory diseases globally. Jingfang Baidu Powder (JF) is a clinically approved traditional Chinese medicine used for treating respiratory infectious diseases. However, its effects and mechanism in the context of ALI remain poorly understood. In this study, we investigated the potential of JF to alleviate ALI by reducing inflammation and necroptosis of lung epithelial cells.

**Methods:**

The effects of JF on ALI were evaluated using an integrative pharmacological strategy. Liquid chromatography-tandem mass spectrometry (LC-MS/MS) was employed to identify the bioactive compounds of JF present in the serum of ALI mice, which are responsible for its therapeutic efficacy in treating ALI. ALI mouse models were established by the nasal drops of Poly (I∶C) over 3 days, followed by, intragastric administration of JF four times. We investigated Changes in lung function, inflammatory cytokines, pathological morphology of lung tissue, mitochondrial indicators, both transcriptomic and metabolomic profiles of the lung tissue, and necroptosis of lung epithelial cells.

**Results:**

JF reduced airway resistance in ALI mice (*p* < 0.01) and respiratory frequency (*p* < 0.05); it lowered the levels of TNF-α in NLF, BALF, and serum (*p* < 0.05 or *p* < 0.01) and the content of IL-6 in NLF (*p* < 0.01), while improving the pathological damage of alveolar epithelial cells and mitochondria in ALI mice. Transcriptomics analysis revealed that JF potentially inhibits necroptosis, with seven constituents, notably hesperidin, identified as the putative active components of JF. Metabolomics analysis demonstrated that JF facilitated pulmonary epithelial cell repair through multifaceted modulation of metabolic pathways. Mechanistic validation indicates that JF can reduce the levels of NEC-related factors LDH and IL-18 in BALF (*p* < 0.05 or *p* < 0.01), as well as decrease the protein levels of p-MLKL and GSDME in lung tissue and the ratio of p-MLKL to MLKL (*p* < 0.05 or *p* < 0.01). Additionally, both JF and hesperidin can reduce NEC in A549 cells (*p* < 0.05 or *p* < 0.01).

**Conclusion:**

JF can ameliorate ALI through various mechanisms, including its anti-inflammatory activity, inhibition of necroptosis, and enhancement of mitochondrial bioenergetics to promote ATP biosynthesis.

## 1 Introduction

Acute lung injury (ALI) and its severe manifestation, acute respiratory distress syndrome (ARDS), represent a significant global health challenge with persistently high mortality rates, particularly among critically ill patients suffering from trauma, sepsis, or infectius diseases worldwide (Long et al., 2022; Zhang et al., 2022). Clinically characterized by refractory hypoxemia, progressive dyspnea, and pulmonary edema, ALI/ARDS pathogenesis involves complex interactions between uncontrolled pulmonary inflammation, disruption of alveolar-capillary barrier integrity, and subsequent cytokine storm-induced organ dysfunction. The devastating impact of these conditions was starkly illustrated during the COVID-19 pandemic, wherein SARS-CoV-2-mediated ARDS accounted for approximately 7 million deaths globally, underscoring the urgent need for effective therapeutic interventions (Lu et al., 2023; Yang et al., 2024). Inhibiting the progression of ALI is considered an effective strategy for preventing ARDS. Despite extensive research, therapeutic advances for ALI remain limited, with no pharmacological interventions demonstrating definitive clinical efficacy to date. Several studies have indicated that certain drugs, including glucocorticoids, can ameliorate ALI (Guo et al., 2023). However, large-scale clinical trials have shown their ineffectiveness in preventing progression to ARDS or reducing long-term mortality in patients who are hypoxic but do not requiring ventilatory support (Lu et al., 2023; Yang et al., 2024). The therapeutic impasse is further compounded by the multifactorial nature of ALI pathophysiology, characterized by a self-amplifying cascade involving immune cell activation, endothelial barrier disrupting, necroptotic alveolar epithelial injury, and cellular energy metabolism dysregulation (Bos and Ware, 2022). Notably, recent systems biology approaches have positioned mitochondrial dysfunction as a central driver of ALI progression, wherein bioenergetic failure through suppressed oxidative phosphorylation, ROS-mediated amplified inflammation and impaired mitophagy-driven quality control collectively perpetuate tissue damage (Pokharel et al., 2024). These emerging mechanisms highlight potential therapeutic targets at the intersection of immunometabolism and cellular homeostasis.

The lack of targeted therapies necessitates innovative approaches that simultaneously address multiple pathogenic cascades. Traditional Chinese medicine (TCM) formulations, with their inherent poly-pharmacological properties, present promising candidates for such multi-target interventions. However, rigorous scientific verification of their mechanisms remains critical. In this context, our study investigates the therapeutic potential of Jingfang Baidu Powder (JF) against ALI, focusing on its potential effects on inflammatory modulation and mitochondrial homeostasis restoration.

**FIGURE 1:**
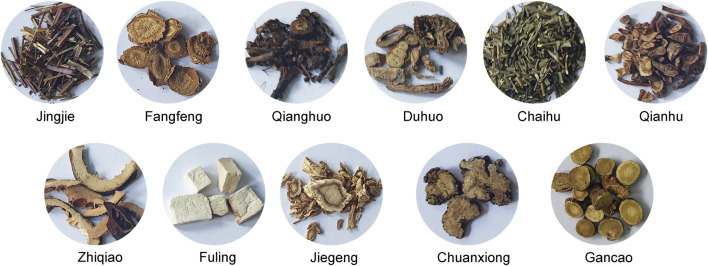
Herbs of JF.

JF is a well-known TCM, extensively prescribed for treating respiratory tract infection (Zhao et al., 2020). It originates from the classic TCM text < *She Sheng Zhong Miao Fang>,* which was authored by Shiche Zhang (Meng et al., 2024). JF consisted of *Schizonepeta tenuifolia* Briq. (jingjie), *Saposhnikovia divaricata* (Turcz.) Schischk. (fangfeng), *Notopterygium incisum* Ting ex H. T. Chang (qianghuo), *Angelica pubescens* Maxim. f. *biserrata* Shan et Yuan (duhuo), *Bupleurum chinense* DC. (chaihu), *Peucedanum praeruptorum* Dunn (qianhu), *Ligusticum chuanxiong* Hort. (chuanxiong), *Citrus aurantium* L. (zhiqiao), *Poria cocos* (Schw.) Wolf. (fuling), *Platycodon grandiflorum* (Jacq.) A. DC. (jiegeng) and *Glycyrrhiza uralensis* Fisch. (gancao) (Figure 1). According to the theory of TCM, JF has a certain potential value of analgesic and antipyretic effect through sweating and dispelling exogenous evils. Clinical reports indicate that JF exhibits anti-inflammatory, anti-viral, and immune regulatory effects (Feng et al., 2022). Furthermore, JF is suitable for use in children, pregnant women, and other populations requiring cautious medication, owing to its minimal side effects. In basic research, Professor Zhang demonstrated that JF granules alleviate bleomycin-induced (Sun X. et al., 2024) ALI by regulating the PI3K/Akt/mTOR signaling pathway (Sun X. et al., 2024). Additionally, JF has also been successfully utilized to treat upper respiratory infections caused by influenza A and has been identified as a potential inhibitor of SARS-CoV-2 (Feng et al., 2020). However, the role of JF in ALI remains poorly understood. The present research aims to elucidate the effects of JF in alleviating ALI and to explore the underlying mechanisms. We analyzed the serum components of JF in a mouse model of ALI, and employed transcriptomics and metabolomics to investigate the material basis and molecular mechanisms by which JF exerts its effects against ALI.

## 2 Materials and methods

### 2.1 Chemicals and regents

Dexamethasone (Dex, LB2269) was obtained from Zhejiang Xianju Pharmaceutical Co., Ltd. MLKL Mouse McAb (66675-1-Ig), DFNA5/GSDME Rabbit PolyAb (13075-1-AP), HRP-conjugated Beta Actin Monoclonal antibody (HRP-66009), HRP-conjugated Affinipure Goat Anti-Rabbit IgG (H + L) (SA00001-2), CoraLite 488-conjugated Goat Anti-Rabbit IgG (H + L), HRP-conjugated Affinipure Goat Anti-Mouse IgG (H + L) (SA00001-1), BCA Protein Assay Kit (PK10026), Hesperidin (CM00691) and Necrostatin-1 (CM00423-) were purchased from Proteintech Group, Inc. Anti-MLKL (phospho S345) antibody (ab196436) was obtained from Abcam Co. Polyinosinic-polycytidylic acid salt (poly (I∶C), PIC, P1530) was purchased from Sigma-Aldrich Trading Co.,Ltd. RIPA lysis buffer (R0010), Protein Phosphatase inhibitor (All-in one, 100×) (P1260), Mounting Medium, antifading (with DAPl) (S2110) and electron microscope fixative (G1102) were purchased from Beijing Solarbio Science & Technology Co., Ltd. Triton X-100 Solution (ST797) and Necroptosis Inducer Kit with TSZ (C1058S) were obtained from Beyotime Biotech Inc commit. CellTiter96® AQueous One Solution Cell Proliferation Assay was obtained from Promega Biotech Co., Ltd. Fetal Bovine Serum (10099141C) and DMEM/F-12 (1:1) (C11330500BT) basic medium were purchased from Grand Island Biological Company.

### 2.2 Preparation of JF

The medicinal materials of JF were obtained from Yunnan Ci Hui Pharmaceutical Co. Ltd. Except for 12 g of gancao, 36 g of each of the other ten medicinal herbs was measured, all materials were soaked in 4500 mL of distilled water for 30 min, the herb mixture was boiled for 20 min to collect the decoction and filtered. The JF water decoction was evaporated by a vacuum concentrator at 55 °C and concentrated to 15.5 mL. The concentrated liquid is freeze-dried, and the powder weight after drying is 6.98 g. After sealing, it is stored in a refrigerator at 4 °C for use.

### 2.3 Animal experiments

C57BL/6J male mice (20–22 g, SPF grade, 8 weeks old) and SD rats (200–220 g, SPF grade, 8 weeks old) were housed in a specific pathogen-free-grade animal facility. The animal studies were conducted with the Guidelines for Animal Experiments of Yunnan University of Chinese Medicine. The study protocol received approval from the Institutional Experimental Animal Ethics Committee of Yunnan University of Traditional Chinese Medicine (No. R-062023125; YNUTCM-XMSS-G-20250016). The mice were housed under a diurnal lighting condition (12/12-h light/dark cycle) for 1 week.

### 2.4 Determination of medicated serum components

16 mice were randomly divided into two groups (n = 8 each): JF and PIC + JF-treated groups, both groups were treated with 0.45 g JF extract/kg (equivalent to four times of the clinical dosage in human, which equivalent dose calculation is based on body surface area). Mice in the PIC + JF-treated group were administered 400 μg of PIC through the right nostril via slow dripping over 3 days to establish the ALI model (Choi et al., 2010). 6 h post-exposure to PIC, the JF and PIC + JF-treated groups received subsequent administrations via gavage for a duration of 4 days 2 h following the last administration, the serum samples were collected and allowed to stand at room temperature for 30 min. Then the samples were centrifuged at 3,500 rpm for 10 min at 4 °C. Subsequently, 1 mL of serum was mixed with 4 mL of pre-cooled methanol and centrifuged at 4 °C at 12,000 rpm for 10 min. The supernatant was then dried using a nitrogen blowing instrument. Following this, 200 μL of pre-cooled methanol was added for re-dissolution, and the mixture was centrifuged at 4 °C at 12,000 rpm for 10 min. The resulting supernatant was used for the identification of small molecular substances.

### 2.5 Study on the effect of JF on ALI model mice

A total of 48 mice were divided into five groups: Control (Con, treated with saline by gavage), PIC (treated with saline by gavage), PIC + dexamethasone (PIC + Dex) (treated with Dex at a dosage of 5 mg/kg by gavage), PIC + JF-H (treated with 0.9 g JF extract/kg by gavage) and PIC + JF-L (treated with 0.45 g JF extract/kg by gavage). Mice in the PIC, PIC + Dex, PIC + JF-H, and PIC + JF-L groups were utilized to establish the ALI model, following the methodology described in Section 2.4. 6 h post-exposure to PIC, JF-treated groups received subsequent administrations at the specified doses for 4 days. The Dex-treated group received administrations prior to the initial modeling during sampling. Meanwhile, the Con and PIC groups were treated with saline. After 2 h after the last administration, the mice were euthanized after anesthesia via intraperitoneal injection of zoletil to harvest bio-samples.

### 2.6 Pulmonary function measure

Mice were anesthetized, and their tracheas were dissected for intubation. Started animal lung function detection system to detect lung function and set parameter: 0.2 mL max stroke volume; 30 cm H_2_O max mouth pressure; 0.75 mL deep inflation max volume; 40 cm H_2_O deep inflation max pressure; 140 breaths/min rate; 0 cm H_2_O peep.

### 2.7 Enzyme linked immunosorbent assay (ELISA)

Serum, nasal lavage fluid (NLF), and bronchoalveolar lavage fluid (BALF) were centrifuged at 3,000 rpm for 10 min. The resulting supernatant was then analyzed for the concentrations of TNF-α, IL-6, LDH and IL-18 using a test kit (Nanjing Jiancheng Bioengineering Institute (Nanjing, China)).

### 2.8 Histopathological analysis

The upper lobes of the left lung of mice were promptly fixed in 4% paraformaldehyde, dehydrated using a gradient of ethanol, and cleared with xylene. The tissue was then embedded in paraffin and sliced them into 4.0 μm thick slices. The slices containing the tissue sections were placed in an oven at 60 °C to dry. Subsequently, the sections were immersed in xylene for dewaxing and rehydrated using a gradient of ethanol. Hematoxylin and eosin (H&E) staining was conducted following the manufacturer’s specifications, and scanned with a scanning microscope (Kunming Naray Technology Co., Ltd., SQS-1000SQS-1000, China).

### 2.9 Transmission electron microscope analysis

The upper lobes of the left lung of mice were quickly fixed using an electron microscope fixative composed of 2.5% glutaraldehyde. The tissues were trimmed to 1 mm^3^ and fixed at 4 °C, then fixed in 1% osmic acid for 1.5 h. Subsequently, the samples were dehydrated using graded concentrations of ethanol and acetone, then, soaked, embedded, trimmed, sliced and stained, and observed under transmission electron microscope.

### 2.10 Transcriptomics analysis

Total RNA was extracted from the three individual lung tissues of Con, PIC, PIC + JF-L (PIC + JF) groups, using TRIzol®Reagent according the manufacturer’s instructions. RNA purification, reverse transcription, library construction and sequencing were performed at Shanghai Majorbio Bio-pharm Biotechnology Co., Ltd. (Shanghai, China). The RNA-seq transcriptome librariy was prepared following Illumina®Stranded mRNA Prep, Ligation (SanDiego, CA) using 1 μg of total RNA. The sequencing library was performed on DNBSEQ-T7platform (PE150) using DNBSEQ-T7RS Reagent Kit (FCLPE150) version3.0. To identify DEGs (differential expression genes) between three groups, the expression level of each transcript was calculated according to the transcripts per million reads (TPM) method. DEGs with |log2FC|≥1 and *p*-value < 0.05, were enriched in GO and KEGG at Bonferroni-corrected *p*-value <0.05 compared with the whole-transcriptome background.

### 2.11 Metabolomics analysis

Lung tissues (the source of bio-samples is the same as 2.10) were extracted by a mixture of methanol, acetonitrile and water (v/v/v, 2:2:1) (1 mL), and then placed for 1 h ultrasonic shaking in ice baths. The mixture was placed at −20 °C for 1 h and centrifuged at 14,000 g for 20 min at 4 °C. The supernatant was collected and dehydrated under vacuum conditions. Metabolomics profiling was analyzed using a UPLC-ESI-Q-Orbitrap-MS system (UHPLC, Shimadzu Nexera X2 LC-30AD, Shimadzu, Japan) coupled with Q-Exactive Plus (Thermo Scientific, San Jose, United States). For liquid chromatography (LC) separation, samples were analyzed using a ACQUITY UPLC® HSS T3 column (2.1 × 100 mm, 1.8 μm) (Waters, Milford, MA, United States). The flow rate was 0.3 mL/min and the mobile phase contained: A: 0.1% FA in water and B: 100% acetonitrile (ACN). The gradient was 0% buffer B for 2 min and was linearly increase to 48% in 4 min, and then up to 100% in 4 min and maintained for 2 min, and then decreased to 0% buffer B in 0.1 min, with 3 min re-equilibration period employed. The electrospray ionization (ESI) with positive-mode and negative mode were applied for MS data acquisition separately. The HESI source conditions were set as follows: Spray Voltage: 3.8 kv (positive) and 3.2 kv (negative); Capillary Temperature: 320 °C; Sheath Gas (nitrogen) flow: 30 arb (arbitrary units); Aux Gas flow: 5 arb; Probe Heater Temp: 350 °C; S-Lens RF Level: 50. The instrument was set to acquire over the m/z range 70–1050 Da for full MS. The full MS scans were acquired at a resolution of 70,000 at m/z 200, and 17,500 at m/z 200 for MS/MS scan. The maximum injection time was set to for 100 m for MS and 50 ms for MS/MS. The isolation window for MS2 was set to 2 m/z and the normalized collision energy (stepped) was set as 20, 30 and 40 for fragmentation. The raw MS data were processed using MS-DIAL for peak alignment, retention time correction and peak area extraction. And then, the PCA and PLS-DA were visualized the global metabolic changes, and utilized to select the differential metabolites based on VIP >1.0 (or |log2FC|≥0.58) combined with one way ANOVA (*p* < 0.05). The KEGG database were used to analyze the related metabolic pathways of differential metabolites.

### 2.12 Tissue immunofluorescence analysis

Quickly fixed the upper lobes of the left lung of mice in 4% paraformaldehyde, then embed them in paraffin and slice them into 3.0 μm thick sections. After dewaxing the addition of a pepsin repair solution, which was incubated at 37 °C for 30 min for antigen retrieval. A 2% Triton X-100 permeabilization solution was then added and incubated at room temperature for 20 min. Tissues were incubated with 5% bovine serum albumin at room temperature for 1 h, followed by overnight incubation at 4 °C with p-MLKL (1:200). Afterward, CoraLite 488-conjugated Goat Anti-Rabbit IgG (H + L) (1:500) was incubated at room temperature in dark for 1 h, and DAPI mounting solution was added for sealing. The tissues were subsequently observed under a fluorescence microscope.

### 2.13 Western blot (WB)

The lung tissues of mice were lysed in RIPA lysis buffer with Protein Phosphatase inhibitor. Protein concentration was measured by BCA Protein Assay Kit. Equal amounts of denatured protein were separated by SDS-PAGE and transferred to PVDF membranes (IPVH00010, Millipore, Billerica, United States). Blocked the membranes with 5% bovine serum albumin or 5% no-fat milk for 2 h, then incubate overnight with the following primary antibody at 4 °C: anti-MLKL (1:2000 dilution), anti-p-MLKL (1:2000 dilution), anti-GSDME (1:2000 dilution), and anti-β-actin (1:2000 dilution). The membranes were then incubated with corresponding secondary antibody for 1 h at room temperature. The bands were visualized using UVP gel imaging analysis system (BOX-F3, Beijing Staples Technology Co., Ltd., China) and quantified and analyzed using ImageJ software.

### 2.14 Molecular docking

To further elucidate the role of JF in mitigating ALI, the components in the medicated serum were molecularly docked with TNFR1 and TRPV1. The 3D structures of the primary ingredients of JF were downloaded from the PubChem platform, while TNFR1 and TRPV1 structures were obtained from the Worldwide Protein Data Bank (https://www.rcsb.org/) in PDB format. The large proteins and components were imported to AutoDockTools for hydrogenation, dehydration, and other pretreatments, followed by docking using AutoDock4. Subsequently, the results were visualized using Discovery Studio 2019.

### 2.15 Cell culture and treatment

Human lung adenocarcinoma cells A549 were obtained from the Beina Chuanglian Biotechnology Co., Ltd. (Beijing, China). A549 cells were cultured in DMEM medium containing 10% (v/v) fetal bovine serum and 1% (v/v) penicillin-streptomycin solution at 37 °C in a 5% CO_2_ atmosphere.

### 2.16 Preparation of JF medicated serum

Male SD rats were randomly divided into a control group and a JF treatment group. The control group received physiological saline via gavage, while the JF group was administered 0.68 g of JF extract (equivalent to four times of the clinical dosage in human)via gavage once daily for 7 consecutive days. 2 h after the final administration, the rats were anesthetized with isoflurane, and blood was collected from the abdominal aorta. The blood samples were placed in EP tubes and allowed to stand at room temperature for 30 min before being centrifuged at 3000 rpm for 10 min at 4 °C. The serum was inactivated in a water bath at 56 °C, aliquoted, and stored at −80 °C.

### 2.17 Cell viability assay

A549 cells in the logarithmic growth phase were seeded at a density of 4 × 10^4^ cells/well in a 96-well culture plate and incubated in a carbon dioxide cell culture incubator. After cell adhesion, the cells were grouped and treated. The experiment was divided into four groups: normal group, model group (NEC), JF-containing serum group (with concentrations of 40%, 20%, 10%, 5%, 2.5%, 1.25%, and 0.625%), and Hes group (with concentrations of 328 μM, 164 μM, 82 μM, 41 μM, 20.5 μM, 10.25 μM, and 5.125 μM). The normal group was maintained under standard culture conditions, while the other groups were treated with a cell necroptosis inducer to establish a cell injury model. Following this, sample working solutions were added for a duration of 24 h. Subsequently, 20 μL of a 5 mg/mL MTS solution was added to each well and incubated at 37 °C for 2 h to ensure complete dissolution. The OD value was then measured at a wavelength of 490 nm using a multifunctional enzyme labeler.

### 2.18 Cellular immunofluorescence analysis

A549 cells were seeded at a density of 1 × 10^5^ cells per well in a 12-well cell culture plate containing glass slides to ensure uniform distribution, and subsequently transferred to a CO_2_ incubator for cultivation. The experiment was divided into five groups: the normal group, the NEC group, the inhibitor group (Necrostatin-1 (NEC-1), 20 µM), the JF-containing serum group (2.5%, 5%), and the Hes group (41 μM, 20.5 µM). Except for the normal group, all other groups were treated with a cell necroptosis inducer to establish a cell injury model, alongside the addition of sample working solutions for 24 h. After 24 h of treatment, the medicated medium was removed from the wells, and the wells were washed with PBS. Subsequently, a cell fixation solution was added for 10 min at room temperature, followed by the addition of a pepsin repair solution, which was incubated at 37 °C for 30 min for antigen retrieval. A 2% Triton X-100 permeabilization solution was then added and incubated at room temperature for 20 min. Cells were incubated with 5% bovine serum albumin at room temperature for 1 h, followed by overnight incubation at 4 °C with p-MLKL (1:200). Afterward, CoraLite 488-conjugated Goat Anti-Rabbit IgG (H + L) (1:500) was incubated at room temperature in dark for 1 h, and DAPI mounting solution was added for sealing. The cells were subsequently observed under a fluorescence microscope, and fluorescent images were captured randomly.

### 2.19 Statistical analysis

All parameters were expressed as means ± SEM. Kruskal–Wallis test and analysis of variance (ANOVA) were performed to compare the means with the control group. Data were analyzed using GraphPad Prism9 software. Statistically significant differences were accepted at *p* < 0.05.

## 3 Results

### 3.1 Compounds of JF in serum of ALI mice

**FIGURE 2:**
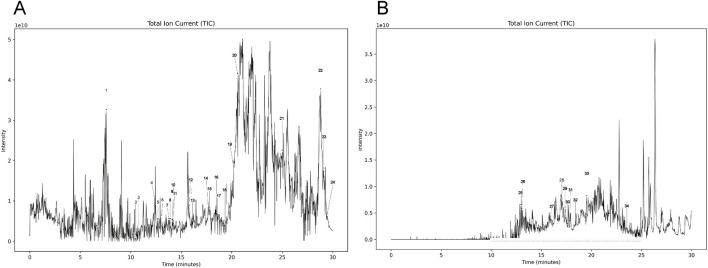
The results of identification of transitional components in medicated serum of ALI model intervened by JF. **(A)** Positive ion mode; **(B)** Negative ion mode.

**FIGURE 3:**
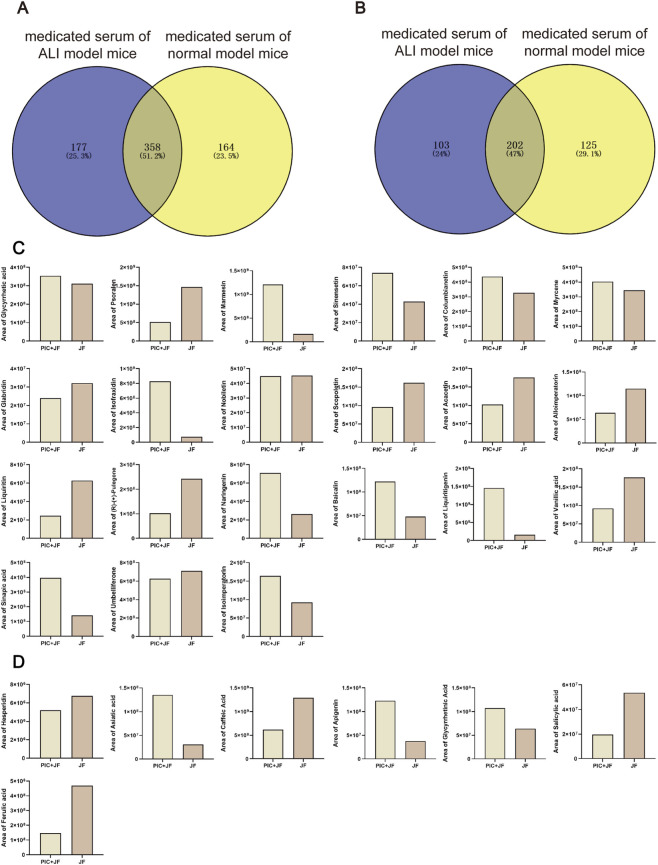
The content changes of the same components in the medicated serum of normal or ALI mice after JF intervention. **(A,C)** Positive ion mode; **(B,D)** Negative ion mode.

The results of identification of the transitional components in medicated serum of ALI model intervened by JF showed that a total of 305 components were detected in the negative ion mode, while 535 components were identified in the positive ion mode (Figure 2). By comparing these findings with existing literature and the TCMSP database, the sources of 34 components were analyzed, including 14 flavonoids, 7 coumarins, 8 terpenoids, 3 phenolic acids, 1 phenylpropanoid component and 1 other class components (Table 1). Among these, 10 components are derived from zhiqiao; 9 from fangfeng; 9 from qianhu, 9 from gancao, 8 from duhuo, 8 from qianghuo, 7 from chuanxiong, 4 from chaihu, 3 from jinjie and 1 from jiegeng. The medicinal materials with the most flavonoids was zhiqiao (6), while fangfeng and qianghuo both contained the most coumarins (6 each). In the medicated serum of normal mice after JF intervention, a total of 327 components were detected in the negative ion mode and 522 components were detected in the positive ion mode. Compared with the medicated serum of ALI mice with that of normal mice, there were 358 identical components in positive ion modes and 202 in negative ion modes. The same components were derived from 28 sources, with the content of 14 components increasing in the serum of ALI mice, 13 components decreasing, and the content of 1 component showing minimal difference (Figure 3).

**TABLE 1:** Identification of transitional components in medicated serum of ALI model intervened by JF.

No.	RT/Min	m/z	Adduct	Identification	Stem from	Classification
1	7.558664	271.0897	[M + H]^+^	Alloimperatorin	fangfeng	Coumarins
2	10.54755	347.1248	[M + Na]^+^	Glabridin	gancao	Flavonoids
3	10.77748	169.0502	[M + H]^+^	Vanillic acid	fangfeng qianhu qianghuo	Phenolic acids
4	12.36994	225.0756	[M + H]^+^	Sinapic acid	chuanxiong	Others
5	12.68684	193.0497	[M + H]^+^	Scopoletin	fangfeng duhuo qianhu chuanxiong (Ren et al., 2007) qianhuo gancao chaihu	Coumarins
6	13.00359	455.0945	[M + Na]^+^	Isovitexin	zhiqiao (Wang et al., 2022)	Flavonoids
7	13.87097	447.0898	[M + H]^+^	Baicalin	chaihu	Flavonoids
8	13.91276	285.0757	[M + H]^+^	Acacetin	jiegeng	Flavonoids
9	14.10298	441.117	[M + Na]^+^	Liquiritin	gancao	Flavonoids
10	14.1255	273.0775	[M + H]^+^	Naringenin	zhiqiao	Flavonoids
11	14.18822	187.0399	[M + H]^+^	Psoralen	fangfeng duhuo qianhu	Flavonoids
12	15.85314	285.0755	[M + H]^+^	Wogonin	fangfeng	Flavonoids
13	16.1288	247.0981	[M + H]^+^	Marmesin	fangfeng duhuo qianhu zhiqiao (Wang et al., 2022) qianghuo	Coumarins
14	17.66541	257.0793	[M + H]^+^	Liquiritigenin	gancao	Flavonoids
15	17.81382	163.0388	[M + H]^+^	Umbelliferone	fangfeng (Li et al., 2017) duhuo qianhu zhiqiao (Wang et al., 2022) qianghuo	Coumarins
16	18.50737	293.0833	[M + Na]^+^	Isoimperatorin	fangfeng duhuo qianhu zhiqiao (Wang et al., 2022) qianghuo	Coumarins
17	18.59222	223.0649	[M + H]^+^	Isofraxidin	fangfeng qianhu (Song et al., 2022) qianghuo (Ma and Yang, 2021)	Coumarins
18	19.35135	403.1383	[M + H]^+^	Nobiletin	zhiqiao	Flavonoids
19	20.19486	373.1277	[M + H]^+^	Sinensetin	zhiqiao (Wang et al., 2022)	Flavonoids
20	20.67813	153.1259	[M + H]^+^	(R)-(+)-Pulegone	jingjie chaihu	Terpenoids
21	23.78942	471.3464	[M + H]^+^	Glycyrrhetic acid	gancao	Terpenoids
22	28.82903	159.1164	[M + Na]^+^	Myrcene	jingjie chaihu chuanxiong zhiqiao	Terpenoids
23	29.102	247.0882	[M + H]^+^	Columbianetin	qianghuo (Ma and Yang, 2021) duhuo (Zhou and Zeng, 2019)	Coumarins
24	29.41642	191.1083	[M + H]^+^	Ligustilide	chuanxiong (Ren et al., 2021)	Terpenoids
25	13.00773	609.1917	[M-H]^-^	Hesperidin	zhiqiao jingjie	Flavonoids
26	13.09256	193.0515	[M-H]^-^	Ferulic acid	qianhu duhuo qianghuo chuanxiong (Ren et al., 2021)	Phenylpropanoids
27	15.88148	137.0652	[M-H]^-^	Salicylic acid	chuanxiong (Ren et al., 2021)	Phenolic acids
28	17.06109	179.0353	[M-H]^-^	Caffeic Acid	duhuo (Zhou and Zeng, 2019) chuanxiong	Phenolic acids
29	17.35511	837.4006	[M-H]^-^	Licoricesaponin G2	gancao	Terpenoids
30	17.60662	269.0457	[M-H]^-^	Apigenin	zhiqiao	Flavonoids
31	17.81761	821.4g023	[M-H]^-^	Glycyrrhizin	gancao (Shakeri et al., 2018)	Terpenoids
32	18.386	255.0669	[M-H]^-^	Isoliquiritigenin	gancao	Flavonoids
33	19.50174	487.3405	[M-H]^-^	Asiatic acid	qianhu	Terpenoids
34	23.53674	469.3346	[M-H]^-^	Glycyrrhetinic Acid	gancao	Terpenoids

The component data without references were from TCMSP, database.

### 3.2 JF restored lung function and reduced cytokine contents in ALI mice

**FIGURE 4:**
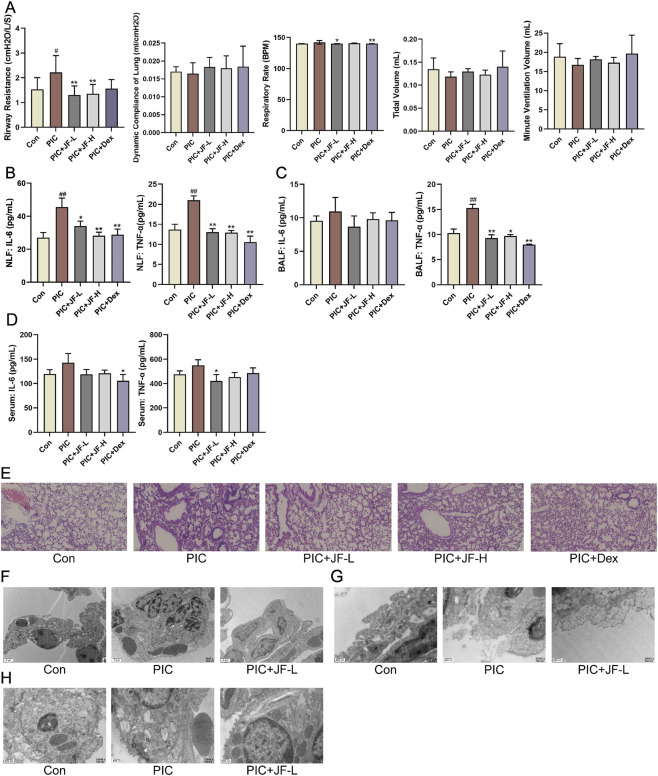
JF restored lung function and reduced cytokine levels in ALI mice. **(A)** Lung function (n = 6); **(B)** Cytokine in NLF (n = 6); **(C)** Cytokine in BALF (n = 6); **(D)** Cytokine in serum (n = 6); **(E)** H&E staining; **(F)** Inflammatory cell infiltration in lung tissue; **(G)** Type I alveolar epithelial cells; **(H)** Type Ⅱ alveolar epithelial cells. Note: vs. Con, ^#^ *p* < 0.05, ^##^ *p* < 0.01; vs. PIC, ^*^ *p* < 0.05, ^**^ *p* < 0.01.

The results of lung function test in mice showed that JF could alleviate airway resistance and respiratory rate (Figure 4A). PIC increased the content of IL-6 and TNF-α in NLF, as well as the TNF-α content in BALF. Notably, JF was shown to reduce the content of IL-6 in NLF, the content of TNF-α in NLF, BALF and serum (Figures 4B–D).

### 3.3 JF improved the pathological damage of alveolar epithelial cells and mitochondria in ALI mice

H&E staining and transmission electron microscopy revealed that the lung tissue of Con group mice exhibited normal morphology, while the lung tissue of the PIC group mice displayed the thickened alveolar walls, infiltration inflammatory cells, necrotic exfoliated alveolar epithelial cells of type I, and type II alveolar epithelial cells osmiophilic multilamellar body vacuoles, mitochondrial membrane rupture. JF and Dex intervention significantly ameliorated the pathological changes in the lung tissue in ALI mice (Figures 4E–H).

### 3.4 RNA-seq analysis of the mechanism by which JF improves ALI

**FIGURE 5:**
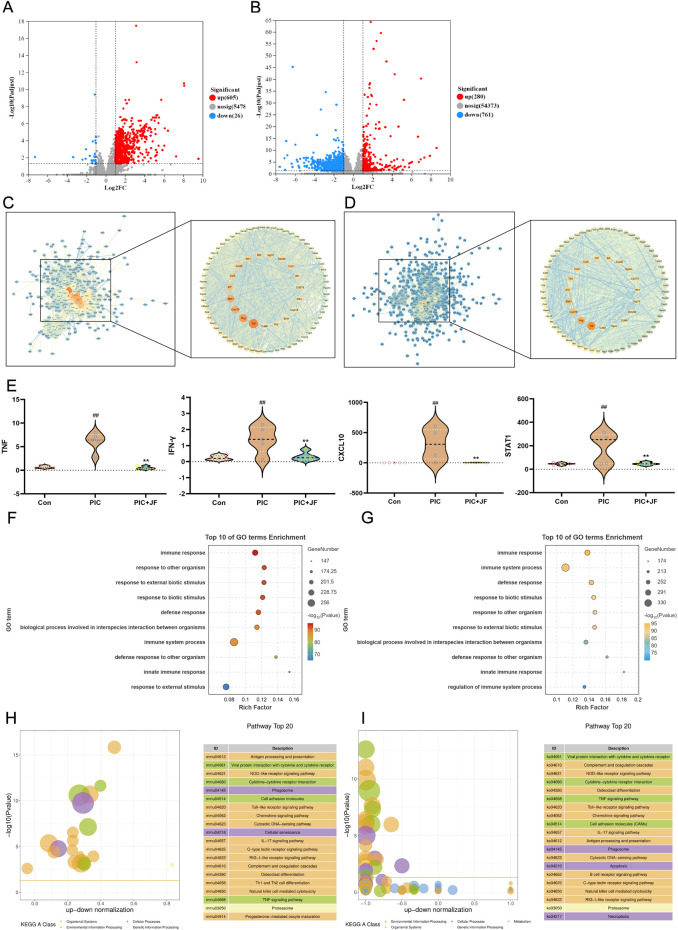
Transcriptome analysis (n = 5). **(A)** Volcano plot of DEGs of PIC vs. Con; **(B)** Volcano plot of DEGs of PIC + JF vs. PIC; **(C)** PPI network of PIC vs. Con; **(D)** PPI network of PIC + JF vs. PIC; **(E)** Violin diagram of core DEGs expression; **(F)** GO enrichment of PIC vs. Con; **(G)** GO enrichment of PIC + JF vs. PIC; **(H)** KEGG enrichment of PIC vs. Con; **(I)** KEGG enrichment of PIC + JF vs. PIC. Note: Here JF group is JF-L group. vs. Con, ^##^ *p* < 0.01; vs. PIC, ^**^ *p* < 0.01.

To explore the molecular basis and mechanism of JF in the treatment of ALI at the genetic level, this study performed transcriptomic sequencing analysis of lung tissues from the Con, PIC group and PIC + JF group in the animal model. According to the results, there were 631 DEGs identified between the PIC and Con groups (605 upregulated and 26 downregulated), and 1041 DEGs between the PIC + JF and PIC groups (280 upregulated and 761 downregulated) (Figures 5A,B). PPI network showed that TNF, IFN-γ, CXCL10 and STAT1 were the core DEGs between the PIC and Con groups, as well as the PIC + JF and PIC groups. Compared to Con group, the mRNA expression of these core DEGs was upregulated in the PIC group, while the PIC + JF intervention significantly downregulated the mRNA expression of these core DEGs compared to the PIC group (Figures 5C–E). All DEGs were functionally enriched, and 10 GO keywords were represented in the bubble plot. The findings revealed that the GO enrichment was primarily associated with immune response, defense response, and response to external biotic stimulus (Figures 5F,G). KEGG enrichment results showed that PIC treatment may affect cell processes such as phagosome and cellular senescence, JF intervention could influence cell processes such as phagosome, apoptosis, and necroptosis (Figures 5H,I).

### 3.5 Non-targeted metabolomics analysis of the mechanism by which JF improves ALI

**FIGURE 6:**
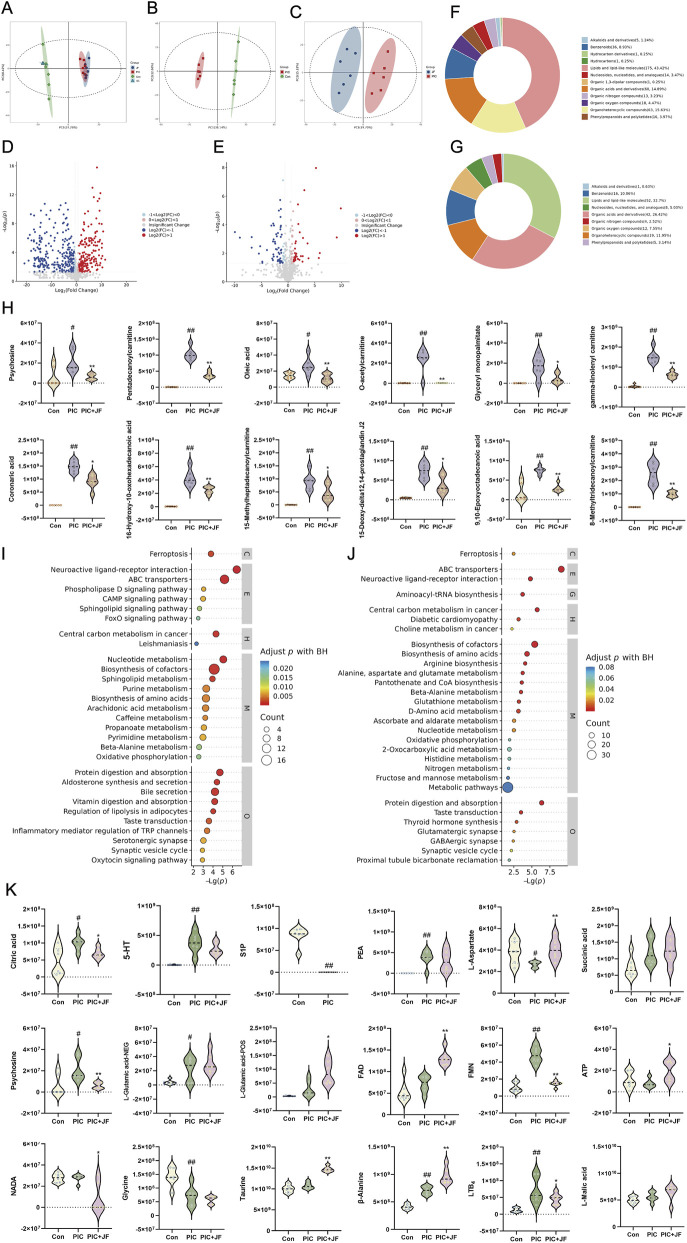
Non-targeted metabolomics (n = 6). **(A)** Quality control; **(B)** PCA analysis of PIC and Con group; **(C)** PCA analysis of PIC + JF and PIC group; **(D)** Volcano plot of DEMs of PIC vs. Con; **(E)** Volcano plot of DEMs of PIC + JF vs. PIC; **(F)** Classification loop diagram of DEMs of PIC vs. Con; **(G)** Classification loop diagram of DEMs of PIC + JF vs. PIC; **(H)** Part of the lipid and lipid-like molecule expression abundance violin diagram; **(I)** KEGG bubble diagram of PIC vs. Con; **(J)** KEGG bubble diagram of PIC + JF vs. PIC; **(K)** Related metabolites in neuroactive ligand-receptor interaction signaling pathway. Note: Here JF group is JF-L group. vs. Con, ^#^ *p* < 0.05, ^##^ *p* < 0.01; vs. PIC, ^*^ *p* < 0.05, ^**^ *p* < 0.01.

PCA analysis was performed on all samples, in the PCA model, the distribution of quality control samples was concentrated, indicating that the sample analysis method had good reproducibility and high stability (Figure 6A). There was a significant difference between the PIC group and the Con group in the PC1 direction, indicating that there was a difference in the metabolic profile of the group. After JF intervention, the distribution of metabolites in mice lung tissue was significantly different from that in the PIC group in the PC1 direction (Figures 6B,C). A total of 421 DEMs were identified between the PIC and Con groups comprising 177 upregulated and 244 downregulated metabolites. Additionally, 170 DEMs (79 upregulated and 91 downregulated) were observed between the PIC + JF and PIC groups (Figures 6D,E). Classification of DEMs revealed that the primary regulated lipids and lipid-like molecules differed between the PIC vs. Con group and PIC + JF vs. PIC groups. The expression abundance of certain lipids and lipid-like molecules increased under PIC intervention, but decreased with JF intervention (Figures 6F–H). KEGG enrichment analysis indicated that PIC mainly affected the signaling pathways such as Neuroactive ligand-receptor interaction, Sphingolipid signaling pathway and Oxidative phosphorylation. In contrast, after JF intervention, the primary pathways affected included Neuroactive ligand-receptor interaction, Arginine biosynthesis and Oxidative phosphorylation (Figures 6I,J). Further analysis of the neuroactive ligand-receptor interaction signaling pathway revealed that JF reduced the expression levels of NADA, Citric acid and Psychosine, while increasing the expression levels of ATP, L-Glutamate, L-Aspartate, β-Alanine and Taurine (Figure 6K).

### 3.6 JF protects ALI by reducing necroptosis

**FIGURE 7:**
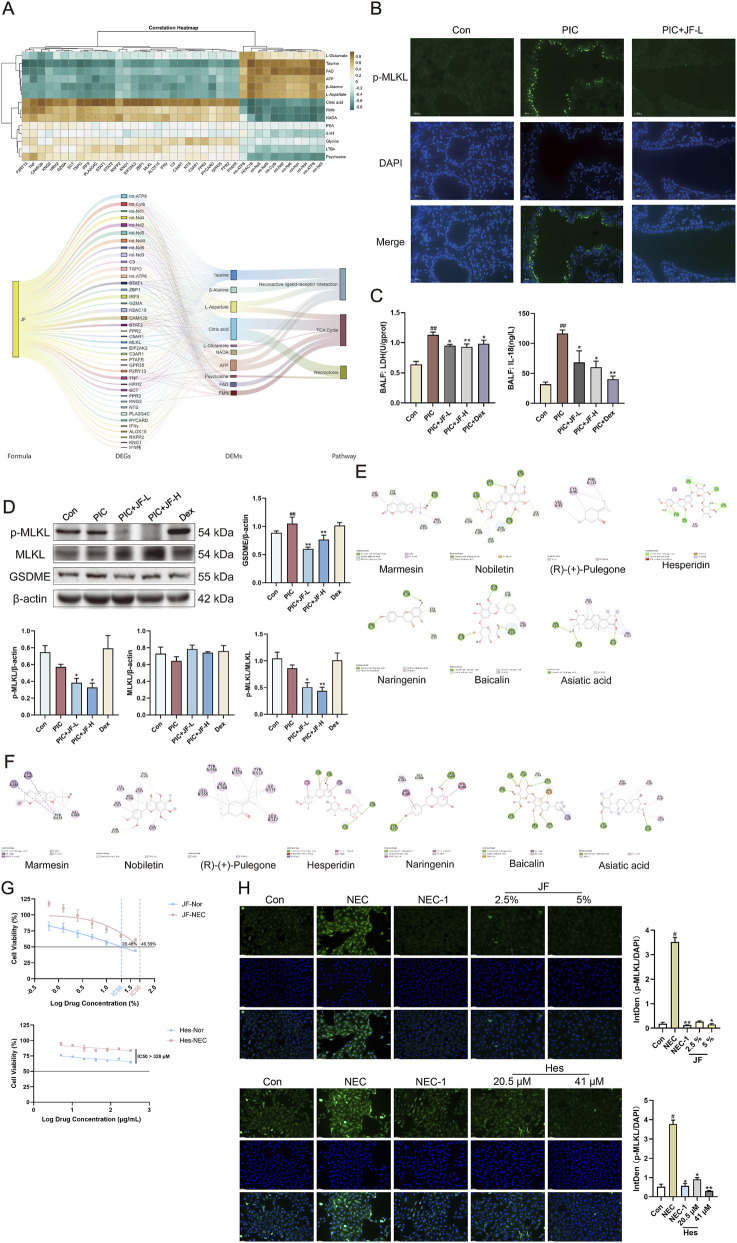
The mechanism of JF alleviating ALI. **(A)** ALI biomarkers regulated by JF and their biological functional heat and network diagram; **(B)** Representative immunofluorescence photomicrographs illustrating p-MLKL (green) in lung tissue. Nuclei (blue) were stained with 4′,6-diamidino-2-phenylindole (DAPI). Bar = 100 μm; **(C)** Cytokine in BALF (n = 6); **(D)** WB analyses were performed to analyze the protein levels of p-MLKL, MLKL, and GSDME (n = 6). **(E)** Molecular docking diagram of serum components of JF and TNFR1; **(F)** Molecular docking diagram of serum components of JF and TRPV1; **(G)** The IC_50_ of JF medicated serum and hesperidin on normal cultured A549 cells and NEC-induced A549 cells (n = 3); **(H)** The effects of JF medicated serum and hesperidin on the fluorescence levels of p-MLKL in A549 cells induced by NEC (n = 3). Note: vs. Con, ^##^ *p* < 0.01; vs. PIC, ^*^ *p* < 0.05, ^**^ *p* < 0.01.

The DEGs and DEMs were utilized to construct a correlation expression heat map, which was subsequently integrated into the “Formula-DEGs-DEMs-pathway” diagram to validate the mechanistic relationship of JF. The role of JF in alleviating ALI was closely related to the regulation of necroptosis, neuroactive ligand-receptor interaction and TCA cycle (Figure 7A). Necroptosis could lead to cell membrane perforation, with p-MLKL and GSDME serving as typical perforins of the cell membrane. Animal immunofluorescence results showed that JF decreased the fluorescence intensity of p-MLKL in lung tissue (Figure 7B), as well as reduced the content of LDH and IL-18 in BALF (Figure 7C), and diminish the protein levels of p-MLKL, GSDME and the conversion rate of p-MLKL/MLKL (Figure 7D). Based on the findings from the omics analysis, it was speculated that TNFR1 and TRPV1 may also be the targets of JF to alleviate ALI. Therefore, the components in the medicated serum were docked with these targets. The results demonstrated that the 7 components represented by baicalin and hesperidin had higher binding affinity with TNFR1 and TRPV1 (Table 2; Figures 7E,F). The animal experiment results showed that necroptosis could occur in lung epithelial cells, so the study was further verified at the cellular level. The experimental results revealed that the IC50 values of JF medicated serum for normal cultured A549 cells and NEC-induced A549 cells were 20.48% and 49.59%, respectively. In contrast, the IC_50_ for Hes in both normal and NEC-induced A549 cells >328 μM (Figure 7G). Treatment with NEC significantly elevated the fluorescence level of p-MLKL in A549 cells. Following treatment with NEC-1, 5% JF medicated serum, as well as 20.5 μM and 42 μM of Hes, there was a significant decrease in the fluorescence level of p-MLKL in A549 cells (Figure 7H).

**TABLE 2:** The docking results of the 7 active compounds of JF to the TNFR1 and TRPV1.

Serum components of JF	Affinity (kcal/mol)	
	TNFR1	TRPV1
Marmesin	−7.9	−7.6
Nobiletin	−8.1	−7.3
(R)-(+)-Pulegone	−5.3	−6.7
Hesperidin	−10.2	−9.0
Naringenin	−8.0	−8.2
Baicalin	−10.3	−8.8
Asiatic acid	−7.8	−8.2

## 4 Discussion

ALI, one of the most common risk factors for ARDS, constitutes a significant global healthcare burden with persistently high morbidity and mortality rates. Despite extensive research, there are no specific pharmacotherapeutic interventions currently exist for ALI management. Therefore, it is crucial to identify effective therapies to reduce lung impairment by better understanding the underlying pathological molecular mechanisms. Recent studies have indicated that ALI is associated with multiple signaling pathways, including NF-κB, MAPK, and NLRP3 inflammasome activation (Madsen et al., 2024). This molecular complexity likely contributes to the limited efficacy of monotargeted therapeutic approaches. Notably, numerous studies suggest the beneficial effects of traditional Chinese medicine (TCM) prescriptions, characterized by their inherent multi-component and polypharmacological properties, which are particularly well-suited for treating ALI (Zhu et al., 2021). Such as Sini decoction and Xuebijing injection have demonstrated promising protective effects against ALI-induced pulmonary damage in preclinical studies through simultaneous modulation of multiple pathological pathways, TCM regimens (Cui et al., 2025).

JF, an ancient Chinese herbal prescription traditionally utilized for treating upper respiratory infections, has demonstrated clinical efficacy in patients with ALI. In this study, we investigated the underlying mechanism of JF in improving ALI. Professor Wang (Wang, 2015) posits that only the ingredients absorbed in serum after oral administration of Chinese medicine prescriptions exert pharmacodynamic effects and are considered pharmacodynamically active. To investigate the pharmacodynamic material basis and mechanism of JF in treating ALI, we conducted serum component identification, transcriptomics and metabolomics following JF administration to ALI mice. We found that JF effectively mitigated lung tissue damage and improved lung function in a PIC-induced mouse ALI model. We identified 34 prototype components of JF in serum samples and found that most of these active components were derived from zhiqiao, fangfeng, qianhu, gancao, duhuo, qianghuo and chuanxiong. Therefore, these medicinal materials may play the same key role in treating ALI.

Upon invasion by respiratory viruses, there is a marked increase in the release of inflammatory cytokines that play a dualistic role. Elevated cytokine production compromises the integrity of the alveolar epithelial barrier, a crucial innate immune defense against pathogen invasion. This barrier, primarily composed of type I (ATI) pneumocytes interspersed with type II (ATII) cells, serves as the first line of defense within the alveoli. It is frequently the initial site of damage during lung injury. Disruption occurs during viral infections, leading to pulmonary edema via increased epithelial permeability and diminished surfactant production due to ATII cell injury (Wang, 2015; Ryabkova et al., 2021). NEC plays a crucial role in the damage of alveolar epithelial cells and the subsequent exacerbation of inflammatory responses (Yang et al., 2022). NEC is a unique form of programmed cell death that occurs independently of cysteine-aspartic protease (caspase) activation. It is characterized by necrotic morphology, including cell swelling, organelle swelling, membrane perforation, followed by cell disintegration and the release of cellular contents. This process leads to extensive infiltration and activation of inflammatory cells, further amplifying the inflammatory response and aggravating lung injury (Ni et al., 2019). TNFR1-driven excessive cell death serves as a powerful driving force for the initiation of inflammation, as it may disrupt biological barriers, resulting in microbial influx and further exacerbating the inflammatory response. Upon recognition of TNF-α by the TNFR1 on the cell membrane, an inflammatory response is triggered. The homotrimerization of TNFR1 leads to the formation of complex I, which can promote cell survival, apoptosis, or necroptosis, depending on the recruitment and ubiquitination of RIPK1. When the ubiquitination of RIPK1 is inhibited, complex I is released and recruits FADD, subsequently forming complex II. Complex II can mediate the activation of Caspase-8, which in turn activates the apoptotic signaling pathway. The FLIP protein, structurally related to but lacking the protease activity of Caspase-8, can form heterodimers that prevent Caspase-8-mediated apoptosis and NEC. The elimination of FADD, Caspase-8, or FLIP results in the spontaneous activation of RIPK3 and mixed lineage kinase domain-like protein (MLKL). In this context, RIPK1 and RIPK3 oligomerize through the RHIM domain to form necrosomes, which serve as conduits for necrotic signaling. Within necrosomes, MLKL is phosphorylated by RIPK3 and subsequently undergoes oligomerization, leading to its localization at the cell membrane. This process culminates in membrane rupture, resulting in the substantial release of intracellular contents and damage-associated molecular patterns (DAMPs) into the surrounding environment. This release triggers inflammation and ultimately leads to necroptotic cell death (Clementi et al., 2021; Vintiñi et al., 2022; Matthay et al., 2019).

**FIGURE 8:**
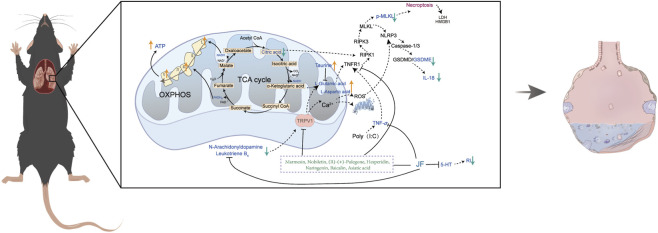
JF attenuated ALI by inhibiting necroptosis and regulating TCA cycle.

Mitochondria, crucial organelles for cellular biosynthesis, energy production, and signal transduction, play a significant role in cellular adaptation to internal environmental homeostasis and the maintenance of normal physiological functions. Recent studies indicate that mitochondrial dysfunction contributes to the development of ALI. Mitochondria are the sites of vital life activities, where the tricarboxylic acid (TCA) cycle and oxidative phosphorylation occur, generating adenosine triphosphate (ATP) to supply energy for the entire cell. The TCA cycle oxidizes carbohydrates, fats, and proteins to produce energy. In eukaryotic systems, the TCA cycle occurs exclusively in the mitochondrial matrix, where aspartate or glutamic acid can be converted into α-ketoglutarate to participate in the cycle. Research indicates that glutamic acid exhibits excitotoxicity, activating the RIPK1/RIPK3/MLKL pathway (Liu et al., 2022). Furthermore, necroptosis can be induced by citrate, an intermediate metabolite of the TCA cycle; when the cycle is inhibited, citrate accumulation may lead to cellular necroptosis. The TCA cycle repeatedly dehydrogenates to produce NADH and FADH2, providing sufficient reducing equivalents for the electron transport chain, which facilitates oxidative phosphorylation and ATP production (Nailwal and Chan, 2019). ATP supplies energy to lung tissues, promoting the proliferation and differentiation of ATII into AECI to repair the alveolar epithelial barrier. During ALI (ALI), there is a disruption of Ca^2+^ homeostasis in the mitochondria of alveolar epithelial cells. Prolonged activation of TRPV1 can induce elevated Ca^2+^ levels, leading to its influx into the mitochondria and causing organelle damage. This effect triggers mitochondrial depolarization, disrupts the membrane potential, and induces the production of reactive oxygen species (ROS) and apoptosis. The disturbance of Ca^2+^ levels and the generation of ROS can activate NLRP3, subsequently recruiting Caspase-1/3 to cleave GSDMD/GSDME, forming membrane pores on the cell membrane and promoting the release of IL-1β and IL-18 (Tonnus et al., 2021). Additionally, dysregulation of lipid metabolism plays a crucial role in the pathogenesis of ALI. For instance, studies demonstrated that imbalanced sphingolipid metabolism contributes to mitochondrial dysfunction and inflammatory amplification in an ALI model, while oxidized phospholipids promote pyroptosis through (Sun N. et al., 2024) the NLRP3 inflammasome pathway (Wang et al., 2025; Sun N. et al., 2024; Yeon et al., 2016). In summary, these studies highlight the therapeutic potential of targeting lipid metabolism reprogramming in ALI. In this study, we found that JF alleviated pulmonary inflammation, improved respiratory function parameters, preserved alveolar epithelial structure, and maintained mitochondrial integrity, thereby mitigating cell damage induced by pathogenic injury. JF and its active component, hesperidin, were shown to reduce NEC in alveolar epithelial cells. These findings establish a novel mechanism through which JF protects against ALI by targeting the NEC-mediated destruction of the alveolar epithelium, thereby highlighting its therapeutic potential for ALI intervention (Figure 8).

Despite the promising findings, our investigation was constrained by several limitations. Comprehensive metabolomics analysis indicated that JF exerts its effects by modulating the TCA cycle and enhancing mitochondrial oxidative phosphorylation, thereby counteracting cell NEC. However, the precise intracellular pathways mediating the anti-NEC actions remains unclear. Future studies should employ single-cell RNA sequencing and spatial metabolomics to dissect cell-type-specific responses and validate the *in vivo* relevance of TRPV1/TNFR1 interactions, which will allow us to better understand the regulatory mechanism of JF on NEC to improve ALI. This study conducts experiments on ALI caused by viral infections, however, it is important to note that numerous triggers for ALI exist, including bacterial infections, chemical irritants, and trauma, which suggests that the research scope may be relatively narrow. Therefore, employing a variety of models to investigate the role and mechanisms of JF is essential. Furthermore, integrating multidisciplinary perspectives from pathogen biology, immunometabolism, and clinical medicine is crucial to elucidate the complex mechanisms involved and to develop stratified intervention strategies that address the challenges posed by ALI diseases.

## Data Availability

The data presented in the study are deposited in the NCBI repository, accession number PRJNA1279349.
