# Supplementary material for: Integrated metabolomic and transcriptomic analysis reveals the effects and mechanisms of Jingfang Baidu powder on acute lung injury
Source: Front Pharmacol. 2026 Jan 9;16:1649883. doi: 10.3389/fphar.2025.1649883 (PMC12827548; doi:10.3389/fphar.2025.1649883)
Supplement: Supplementary file 1 [file Supplementaryfile1.docx]

Supplementary Material

# Supplementary Figure


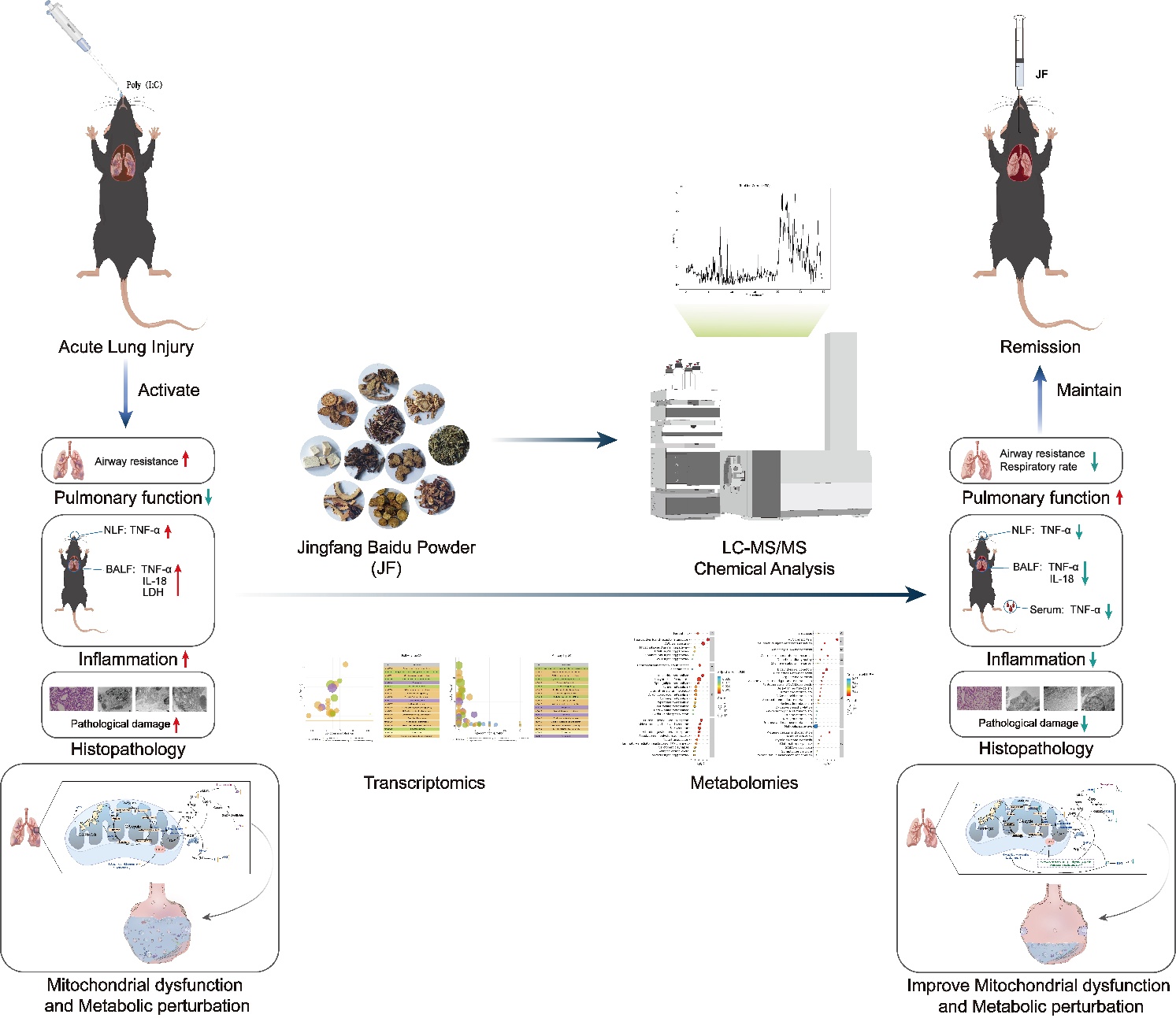


Figure1. graph summarization

# Supplementary Table

Table 1. The content changes of the same components in the medicated serum of normal or ALI mice after JF intervention

| No. | Adduct | Identification | Area | | | Stem from |
| --- | --- | --- | --- | --- | --- | --- |
|  |  |  | PIC + JF | JF | ALI/Normal |  |
| 1 | [M+H]^+^ | Glycyrrhetic acid | 353541184 | 310818272 | ↑ | gancao |
| 2 | [M+H]^+^ | Psoralen | 511650528 | 1462966400 | ↓ | fangfeng  duhuo  qianhu |
| 3 | [M+H]^+^ | Marmesin | 1207715456 | 164030128 | ↑ | fangfeng  duhuo  qianhu  zhiqiao  qianghuo |
| 4 | [M+H]^+^ | Sinensetin | 73827200 | 42712768 | ↑ | zhiqiao |
| 5 | [M+H]^+^ | Columbianetin | 437034944 | 326617664 | ↑ | qianghuo  duhuo |
| 6 | [M+Na]^+^ | Myrcene | 403076384 | 343736448 | ↑ | jingjie  chaihu  chuanxiong  zhiqiao |
| 7 | [M+Na]^+^ | Glabridin | 23897192 | 32080000 | ↓ | gancao |
| 8 | [M+H]^+^ | Isofraxidin | 827898816 | 74844280 | ↑ | fangfeng  qianhu  qianghuo |
| 9 | [M+H]^+^ | Nobiletin | 44952684 | 45185840 | - | zhiqiao |
| 10 | [M+H]^+^ | Scopoletin | 96161680 | 161672224 | ↓ | fangfeng  duhuo  qianhu  chuanxiong  qianghuo  gancao  chaihu |
| 11 | [M+H]^+^ | Acacetin | 102648720 | 176136944 | ↓ | jiegeng |
| 12 | [M+H]^+^ | Alloimperatorin | 63801156 | 114928480 | ↓ | fangfeng |
| 13 | [M+H]^+^ | Liquiritin | 24390952 | 62699816 | ↓ | gancao |
| 14 | [M+H]^+^ | (R)-(+)-Pulegone | 101824128 | 242736576 | ↓ | jingjie  chaihu |
| 15 | [M+H]^+^ | Naringenin | 709847936 | 264091888 | ↑ | zhiqiao |
| 16 | [M+H]^+^ | Baicalin | 122206368 | 47815716 | ↑ | chaihu |
| 17 | [M+H]^+^ | Liquiritigenin | 1457780224 | 156001888 | ↑ | gancao |
| 18 | [M+H]^+^ | Vanillic acid | 92246136 | 176498624 | ↓ | fangfeng  qianhu  qianghuo |
| 19 | [M+H]^+^ | Sinapic acid | 396554880 | 142081216 | ↑ | chuanxiong |
| 20 | [M+H]^+^ | Umbelliferone | 6253238784 | 7100087808 | ↓ | fangfeng  duhuo  qianhu  zhiqiao  qianghuo |
| 21 | [M+H]^+^ | Isoimperatorin | 163897280 | 92387296 | ↑ | fangfeng  duhuo  qianhu  zhiqiao  qianghuo |
| 22 | [M-H]^-^ | Hesperidin | 5194992 | 6748500 | ↓ | zhiqiao  jingjie |
| 23 | [M-H]^-^ | Asiatic acid | 135165008 | 30581842 | ↑ | qianhu |
| 24 | [M-H]^-^ | Caffeic Acid | 616000512 | 1290250368 | ↓ | duhuo  chuanxiong |
| 25 | [M-H]^-^ | Apigenin | 122739728 | 37436024 | ↑ | zhiqiao |
| 26 | [M-H]^-^ | Glycyrrhetinic Acid | 447452512 | 85383232 | ↑ | gancao |
| 27 | [M-H]^-^ | Salicylic acid | 19582182 | 53560060 | ↓ | chuanxiong |
| 28 | [M-H]^-^ | Ferulic acid | 146487968 | 469441120 | ↓ | qianhu  duhuo  qianghuo  chuanxiong |

Table 2. Metabolite Information

| No. | Metabolites | PIC *vs* Con | | | PIC+JF *vs* PIC | | |
| --- | --- | --- | --- | --- | --- | --- | --- |
|  |  | FC | VIP | p-value | FC | VIP | p-value |
| 1 | Psychosine | 3.26 | 0.97 | <0.05 | 0.29 | 1.64 | <0.01 |
| 2 | Pentadecanoylcarnitine | 189.81 | 1.57 | <0.001 | 0.37 | 2.13 | <0.001 |
| 3 | Oleic acid | 1.83 | 1.03 | <0.05 | 0.41 | 1.66 | <0.01 |
| 4 | O-acetylcarnitine | 179.36 | 1.35 | <0.001 | 0.009 | 1.91 | <0.001 |
| 5 | Glyceryl monopalmitate263.86 | 263.86 | 1.27 | <0.01 | 0.27 | 1.49 | <0.05 |
| 6 | gamma-linolenyl carnitine | 32.15 | 1.55 | <0.001 | 0.40 | 2.04 | <0.001 |
| 7 | Coronaric acid | 1064.99 | 1.60 | <0.001 | 0.60 | 1.81 | <0.01 |
| 8 | 16-Hydroxy-10-oxohexadecanoic acid | 120.6 | 1.52 | <0.001 | 0.57 | 1.69 | <0.01 |
| 9 | 15-Methylheptadecanoylcarnitine | 1997.66 | 1.46 | <0.001 | 0.51 | 1.39 | <0.05 |
| 10 | 15-Deoxy-delta12,14-prostaglandin J2 | 15.46 | 1.53 | <0.001 | 0.51 | 1.58 | <0.05 |
| 11 | 9,10-Epoxyoctadecanoic acid | 4.03 | 1.39 | <0.001 | 0.39 | 2.17 | <0.001 |
| 12 | 8-Methyltridecanoylcarnitine | 303.00 | 1.51 | <0.001 | 0.39 | 1.94 | <0.001 |
| 13 | Citric acid | 2.12 | 1.09 | <0.05 | 0.69 | 1.56 | <0.05 |
| 14 | 5-HT | 117.94 | 1.38 | <0.001 | 0.72 | 0.89 | >0.05 |
| 15 | Sphingosine 1-phosphate | 0.002 | 1.55 | <0.001 | - | - | - |
| 16 | PEA | 2819.369 | 1.34 | <0.01 | 0.82 | 0.43 | >0.05 |
| 17 | L-Aspartate | 0.75 | 0.94 | >0.05 | 1.94 | 2.15 | <0.001 |
| 18 | Succinic acid | 1.63 | 0.95 | <0.05 | 1.03 | 0.06 | >0.05 |
| 19 | Psychosine | 3.26 | 0.97 | <0.05 | 0.29 | 1.64 | <0.01 |
| 20 | L-Glutamic acid-POS | 8.48 | 0.90 | >0.05 | 3.10 | 1.56 | <0.05 |
| 21 | L-Glutamic acid-NEG | 6.00 | 1.08 | <0.05 | 1.34 | 0.58 | >0.05 |
| 22 | FAD | 1.31 | 0.62 | >0.05 | 1.85 | 2.02 | <0.001 |
| 23 | FMN | 4.67 | 1.51 | <0.001 | 0.30 | 2.15 | <0.001 |
| 24 | ATP | 0.72 | 0.43 | >0.05 | 2.53 | 1.51 | <0.05 |
| 25 | NADA | 1.00 | 0.02 | >0.05 | 0.33 | 1.66 | <0.05 |
| 26 | Glycine | 0.51 | 1.2 | <0.01 | 0.80 | 0.66 | >0.05 |
| 27 | Taurine | 1.07 | 0.70 | >0.05 | 1.37 | 2.20 | <0.001 |
| 28 | β-Alanine | 1.73 | 1.51 | <0.001 | 1.37 | 1.80 | <0.01 |
| 29 | LTB_4_ | 5.67 | 1.23 | <0.01 | 0.84 | 0.35 | >0.05 |
| 30 | L-Malic acid | 1.11 | 0.62 | >0.05 | 1.14 | 0.78 | >0.05 |
